# Pulmonary hypertension in infants with congenital heart defects: are leukotrienes involved?

**DOI:** 10.1080/09629359791451

**Published:** 1997-12

**Authors:** A. Serraf, J-P. Gascard, J. Bruniaux, C. Labat, C. Planche, C. Brink

**Affiliations:** 1 Service de Chirurgie et Réanimation Cardiaques Pédiatriques Centre Chirurgical Marie-Lannelongue 133, avenue de la Résistance Le Plessis-Robinson 92350 France; 2 CNRS ERS 566 Centre Chirurgical Marie-Lannelongue 133, avenue de la Résistance Le Plessis-Robinson 92350 USA

## Abstract

The circulating levels of leukotriene E_4_ in infants with congenital heart defects, increased pulmonary blood flow and pulmonary arterial hypertension, were determined and compared with infants with decreased pulmonary blood flow (Tetralogy of Fallot). There was no correlation (*r*=0.38) between the pulmonary arterial pressure (56 ± 4 mmHg) and the leukotriene E_4_ levels (1.37 ± 0.67 ng/ml blood) measured in peripheral blood samples from the hypertensive group prior to surgery. There was considerable variation in the detectable leukotriene E_4_ levels in blood samples from different patients. The levels detected in the blood samples between the two groups of patients was similar. These data suggest that neither the surgical repair during cardiopulmonary bypass nor the pulmonary hypertension appeared to modify the leukotriene E_4_ blood levels in the small number of patients studied.


Mediators of Inflammation, **6**, 323–326 (1997)

### Pulmonary hypertension in infants with congenital heart defects: are leukotrienes involved?

A. Serraf[1], J-P. Gascard[2], J. Bruniaux[1], C. Labat[2], C. Planche[1] and C. Brink[2,CA]

[1]Service de Chirurgie et Réanimation Cardiaques Pédiatriques, and [2]CNRS ERS 566, Centre Chirurgical Marie-Lannelongue, 133, avenue de la Résistance, 92350 Le Plessis-Robinson, France

[CA]Corresponding Author
Tel: (+33) 1 40 94 28 00 ext. 3015
Fax: (+33) 1 46 30 12 08
Email: brink@pratique.fr

**The circulating levels of leukotriene $E_4$ in infants with congenital heart defects, increased pulmonary blood flow and pulmonary arterial hypertension, were determined and compared with infants with decreased pulmonary blood flow (Tetralogy of Fallot). There was no correlation ($r = 0{\cdot}38$) between the pulmonary arterial pressure ($56 \pm 4$ mmHg) and the leukotriene $E_4$ levels ($1{\cdot}37 \pm 0{\cdot}67$ ng/ml blood) measured in peripheral blood samples from the hypertensive group prior to surgery. There was considerable variation in the detectable leukotriene $E_4$ levels in blood samples from different patients. The levels detected in the blood samples between the two groups of patients was similar. These data suggest that neither the surgical repair during cardiopulmonary bypass nor the pulmonary hypertension appeared to modify the leukotriene $E_4$ blood levels in the small number of patients studied.**

**Key words:** Pulmonary hypertension, Cardiovascular surgery, Congenital heart defects, Leukotrienes, Extracorporeal circulation

### Introduction

The hypothesis that metabolites of the arachidonic acid cascade, specifically products of the 5-lipoxygenase pathway, may be involved in pulmonary hypertension was based on two lines of evidence. First, injection of cysteinyl-leukotrienes significantly increased pulmonary vascular resistance in neonatal lambs[1] as well as in mature guinea-pigs and rats.[2] Furthermore, in the monkey, transient pulmonary hypertension was also observed following leukotriene administration.[3] The second line of evidence was provided by Stenmark and coworkers[4] who detected the presence of cysteinyl-leukotrienes in newborns with persistent pulmonary hypertension. These latter observations were based on analysis of bronchoalveolar lavage fluid derived from infants with pulmonary hypertension. Such data suggested indirectly that cysteinyl-leukotrienes which are potent vasoconstrictor agents in the human lung[5] may be associated with elevated pulmonary arterial pressure in children.

Previous surgical reports have shown that there are acute transitory episodes of increased pulmonary arterial pressure in patients following open heart surgery for correction of congenital heart lesions.[6] Unfortunately, the circulating levels of leukotrienes in this subset of patients is not known. In this study, the levels of $LTE_4$ in blood samples derived from infants with congenital heart defects, increased pulmonary blood flow and pulmonary hypertension were determined during the surgical intervention for correction of the heart defect and compared with infants with decreased pulmonary blood flow. Since $LTE_4$ is known to be produced by the human lung *in vitro*[7,8] and is a stable product of the 5-lipoxygenase pathway, the aim of the present study was to establish whether or not circulating $LTE_4$ was correlated with pulmonary arterial pressure and to determine whether the human lung *in situ* released this 5-lipoxygenase pathway metabolite.

### Methods

### Subjects

Eighteen patients with congenital heart defects (2–25 months of age) were studied. Six were diagnosed to have regular form of Tetralogy of Fallot, seven patients had complete atrioventricular septal defect, three had truncus arteriosus and two had ventricular septal defect. All patients with pulmonary hypertension ($n = 12$) presented with heart failure despite medical therapy support. None of them received medication that could interfere with the arachidonic acid cascade. Haemodynamic measurements

© 1997 Rapid Science Publishers

(pulmonary, systemic and atrial pressures) were continuously recorded for each patient using the Hewlett Packard 78354A monitor. Open heart repair was performed with the aid of hypothermic cardiopulmonary bypass. Aortic cross-clamping with injection of blood cardioplegia was used during the intracardiac repair. The mean duration of the cardiopulmonary bypass in Fallot patients was $69 \pm 4$ min and $114 \pm 9$ min in pulmonary hypertension subjects. None of the patients had cardiac repair under deep hypothermic circulatory arrest. After sternal and pericardial opening, blood samples were drawn from the main pulmonary artery and the left atrium. Surgery was then conducted as usual. Prior to weaning from cardiopulmonary bypass, monitoring lines (Seldicath 3 Fr. Plastimed) were introduced in the left atrial cavity and in the main pulmonary artery. Whilst in the intensive care unit, blood samples were collected from the patients via these catheter lines.

### Leukotriene $E_4$ measurements

All blood samples (0.5 ml) were collected directly in tubes containing methanol at the beginning (Start) of extracorporeal circulation (ECC), 5 min after lung reperfusion (RP) and at the end of ECC. The tubes were stored overnight at –20°C. The samples were then thawed, vortexed and centrifuged (5000 rpm for 20 min at 4°C). The supernatant was then removed and added to tubes containing 40 ml of methanol at 10% and this mixture was then passed through a column (Sep-Pak C-18). The extracts containing lipids were collected on 3 ml of methanol. These samples were then separated into equal volume aliquots and dried using a Speed-Vac evaporator. The residue was dissolved in mobile phase solution of HPLC (acetonitrile/water/acetic acid at pH 5.6). This solution (20 µl) was injected into an HPLC (Waters) for separation. The samples were collected and quantification was performed by EIA (Stallergens, Fresnes, France).

Due to the complexity of the surgical intervention, internal standards for $LTE_4$ were not performed. However, using a blood sample obtained prior to surgery in one infant the analytic recoveries for standard $LTE_4$ were 78% for 0·5 ng/ml and 82% for 1 ng/ml, demonstrating that collection of blood samples in tubes containing methanol permitted adequate recoveries of $LTE_4$. The range of $LTE_4$ levels detected in the peripheral blood samples was 0 to 0·76 ng/ml (Fallot patients) and 0 to 7·2 ng/ml (pulmonary hypertension patients).

### Statistical analysis

Results are expressed as means ± SEM. Since the number of patients studied was limited and the $LTE_4$ range was large, no statistical analysis was performed on the data. However, the data for each patient are presented.

### Results

The pulmonary arterial pressure and the quantities of $LTE_4$ detected in peripheral blood samples derived from patients without (Fallot) and with pulmonary hypertension (PH) prior to surgery are shown in Table 1. There was no correlation between the elevated preoperative pulmonary arterial pressure and the levels of circulating $LTE_4$ detected in the peripheral blood samples ($r = 0{\cdot}38$). The $LTE_4$ levels detected in the infants during the course of the surgical intervention are presented in Tables 2 and 3 as well as in Fig. 1. The $LTE_4$ levels measured in blood samples derived from the pulmonary artery for the different patients (Fallot vs. PH) were similar. Values obtained in the samples from the left atrium in these two groups of patients were also similar. In five patients (PH) who were studied over the course of 12 h the $LTE_4$ levels were not altered when compared with those data obtained at the beginning of ECC.

In five of the 18 patients examined (one Fallot and four PH), the $LTE_4$ levels were below the threshold level of detection. Of the 13 patients where $LTE_4$ levels were detectable, eight subjects exhibited higher $LTE_4$ levels in blood samples derived from the left atrium than those levels detected in blood samples from the pulmonary artery during the lung reperfusion period. In two subjects, the $LTE_4$ levels in the left atrium were lower than those measured in the pulmonary artery blood samples. In two patients no samples were obtained and in another patient the values were the same. In addition, the total quantities of $LTE_4$ detected during the surgical intervention were: Fallot,

**Table 1.** Pulmonary arterial pressure and circulating leukotriene $E_4$ levels in infant patients

| Patients | Age (months) | (*n*) | PAP (mmHg) | $LTE_4$ (ng/ml) |
|---|---|---|---|---|
| Fallot | 11.9 ± 3.2 | 6 | NM | 0.44 ± 0.12 |
| PH | 8.5 ± 1.6 | 12 | 56 ± 4 | 1.37 ± 0.67 |

Values are means ± SEM from the number of patients studied (*n*). Age at time of surgery. PH = pulmonary hypertension; PAP = pulmonary arterial pressure (prior to surgery) and NM = not measured. The $LTE_4$ levels were determined in peripheral blood samples prior to surgery.

**Table 2**. $LTE_4$ levels (pg/ml) detected in blood samples obtained from infants (Fallot)

| Patients | Age (months) | Peripheral blood | ECC | | | | | |
|---|---|---|---|---|---|---|---|---|
| | | | Start | | Lung reperfusion | | End | |
| | | | PA | LA | PA | LA | PA | LA |
| 1 | 8 | 590 | 660 | 750 | 432 | 470 | 327 | 440 |
| 2 | 3.2 | 366 | 421 | 471 | 2010 | 1850 | 582 | 1880 |
| 3 | 15.8 | 700 | 1040 | 565 | 525 | 780 | 635 | 665 |
| 4 | 25.7 | 755 | 835 | 765 | 500 | 1105 | 1170 | 1320 |
| 5 | 10.4 | 0 | 0 | 0 | 0 | 0 | 0 | 0 |
| 6 | 8.5 | 246 | 224 | 632 | NP | 445 | 491 | 523 |

PA = pulmonary artery; LA = left atrium; 0 = below level of assay detection; NP = no sample; ECC = extracorporeal circulation; months = age at surgery.

**Table 3**. $LTE_4$ levels (pg/ml) detected in blood samples obtained from infants (PH)

| Patients | Age (months) | PAP (mmHg) | Peripheral blood | ECC | | | | | |
|---|---|---|---|---|---|---|---|---|---|
| | | | | Start | | Lung reperfusion | | End | |
| | | | | PA | LA | PA | LA | PA | PA |
| 1 | 13 | 83 | 406 | 188 | 746 | 149 | 340 | 138 | 253 |
| 2 | 9 | 60 | 630 | 312 | 1180 | 550 | 542 | 551 | 522 |
| 3 | 7.2 | 52 | 0 | 0 | 0 | 0 | 0 | 0 | 0 |
| 4 | 12 | 48 | 0 | 0 | 0 | 0 | 0 | 0 | 0 |
| 5 | 3.7 | NM | 155 | 209 | 603 | NP | NP | 161 | 681 |
| 6 | 15 | 70 | 570 | 1086 | 799 | 413 | 566 | NP | NP |
| 7 | 3.8 | 67 | 5090 | 6679 | 1610 | 7158 | 2317 | 2517 | 1389 |
| 8 | 4.8 | NM | 1217 | 244 | 1743 | 570 | 1832 | 206 | 1404 |
| 9 | 2.7 | 70 | 7172 | 5427 | NP | 532 | 6850 | 4350 | 5920 |
| 10 | 20.5 | 47 | 0 | 0 | 0 | 0 | 0 | 0 | 0 |
| 11 | 2.4 | 40 | 1170 | 1200 | 1310 | 801 | 1370 | 270 | 1500 |
| 12 | 7.8 | 50 | 0 | 0 | 0 | 0 | 0 | 0 | 0 |

PA = pulmonary artery; LA = left atrium; 0 = below level of assay detection; NP = no sample; ECC = extracorporeal circulation; NM = not measured; PAP = pulmonary arterial pressure (prior to surgery); months = age at surgery.

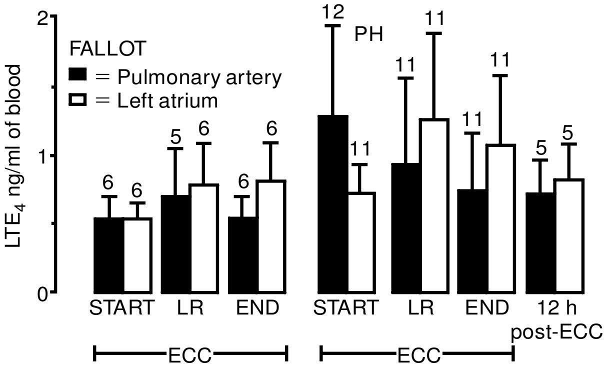

FIG. 1. Circulating leukotriene $E_4$ levels in infants during open heart surgery. Measurements of $LTE_4$ were performed in blood samples obtained during surgery in a group of infants without (Fallot) and with pulmonary hypertension (PH). Blood samples were derived from either the pulmonary artery or the left atrium at the beginning (START) of extracorporeal circulation (ECC), 5 min after lung reperfusion (LR) and at the END of ECC. In a limited number of subjects ($n = 5$) measurements were made at 12 h post-ECC. Values are means ± SEM and the number of infants studied is presented above each bar.

0.64 ± 0.17 ng/ml blood; and PH, 1.03 ± 0.43 ng/ml blood.

### Discussion

While $LTE_4$ was detected in blood samples from infants (72%) an increase in the circulating levels was not observed in those patients with pulmonary hypertension. These data, derived from a limited number of patients, suggested that neither the surgical intervention nor the pulmonary hypertension were associated with an alteration in $LTE_4$ blood levels.

There was considerable variation in the $LTE_4$ levels detected in blood samples obtained from neonates with or without pulmonary hypertension. The range in the quantities of $LTE_4$ detected was similar to those reported by other investigators when this metabolite was measured in either urine,[9–11] bronchoalveolar lavage[12] or blood samples[13] from patients with different clinical pathologies. The reasons for these variations are presently unknown but are probably independent of the pathophysiological condition, since control subjects (Fallot) exhibit a similar range in $LTE_4$ values, when compared with values obtained in infants with pulmonary hypertension. Furthermore, the $LTE_4$ levels detected were not correlated with the high pulmonary arterial pressure observed in PH infants. These data are similar to a previous report in adult pulmonary hypertensive patients where other metabolites of the arachidonic acid cascade were measured.[14] These authors found no correlation between the pulmonary arterial pressure and thromboxane ($TxB_2$) levels in urine samples. These data suggest that the circulating potent vasoconstrictor metabolites of the arachidonic acid pathway ($TxA_2$ and $LTE_4$) which are detected in pulmonary hypertension may not be related to the *in vivo* pressure modulations reported in the pulmonary artery.

The low levels of $LTE_4$ (28% of patients, undetected) and the interpatient variation will now require further investigation. First, there may be a preferential metabolism of arachidonic acid in different individuals. A significant modification in the production/removal equilibrium of the 5-lipoxygenase metabolites could result in alterations in detection of the more stable metabolite ($LTE_4$). Therefore, the measurement of only $LTE_4$ in the biological samples may not be an appropriate index of the 5-lipoxygenase pathway activity in all patients. Unfortunately, no data are available concerning $LTC_4$ and $LTD_4$ levels in biological samples obtained from subjects where no $LTE_4$ was detected. The considerable variation in the levels of $LTE_4$ between patients may also be related to the way $LTE_4$ is bound in the circulation. Unfortunately, little is known about the fixation of cysteinyl-leukotrienes in the human circulation. Few studies have been published dealing with the quantitation of leukotrienes in human blood samples and analysis has been essentially based on results derived from human plasma.[15] Finally, an increase in quantities of leukotrienes may occur in the tissue compartment rather than in the circulation. Indeed, previous data have shown that following an instillation of LT in the rat lung only a small percentage could be detected in the lavage fluid[16] suggesting that the lung tissue rather than the lung liquid was the dominant compartment. Whether or not lung tissue from pulmonary hypertensive patients exhibits elevated levels of $LTE_4$ has not been reported. In order to adequately understand the role of these potent inflammatory mediators in pulmonary hypertension these issues will have to be addressed not only in relation to this disease but also other pathologies where leukotrienes have been implicated.

### References

1. Schreiber MD, Heymann MA, Soifer SJ. The differential effects of leukotriene $C_4$ and $D_4$ on the pulmonary and systemic circulations on newborn lambs. *Pediatr Res* 1987; **21**: 176–182.
2. Berkowitz BA, Zabko-Potapovich B, Valocik R, Gleason JG. Effects of the leukotrienes on the vasculature and blood pressure of different species. *J Pharmocol Exp Ther* 1983; **229**: 105–112.
3. Smedegard G, Hedqvist P, Dahlen SE, Revenas B, Hammarstrom S, Samuelsson B. Leukotriene $C_4$ affects pulmonary and cardiovascular dynamics in monkey. *Nature* 1982; **295**: 327–329.
4. Stenmark KR, James SL, Voelkel NF, Norbert F, Toews WH, Reeves JT, Murphy RC. Leukotriene $C_4$ and $D_4$ in neonates with hypoxemia and pulmonary hypertension. *N Engl J Med* 1983; **309**: 77–80.
5. Labat C, Ortiz JL, Norel X, Gorenne I, Verley J, Abram TS, Cuthbert NJ, Tudhope SR, Norman P, Gardiner PJ, Brink C. A second cysteinyl leukotriene receptor in human lung. *J Pharmacol Exp Ther* 1992; **63**: 800–805.
6. Serraf A, Bruniaux J, Lacourt-Gayet F, Chambran P, Binet JP, Lecronier G, Demontoux S, Planché C. Obstructed total anomalous pulmonary venous return. *J Thorac Cardiovasc Surg* 1991; **101**: 601–606.
7. Kumlin M, Dahlén S-E. Characteristics of formation and further metabolism of leukotrienes in the chopped human lung. *Biochimica et Biophysica Acta* 1990; **1044**: 201–210.
8. Gorenne I, Labat C, Gascard JP, Norel X, Müller-Peddinghaus R, Mohrs KH, Taylor WA, Gardiner PJ, Brink C. ®-2-[4-(Quinolin-2-yl-methoxy)-phenyl-2-cyclopentyl Acetic acid] (BAY x1005) a potent leukotriene synthesis inhibitor: effects on anti-IgE challenge in human airways. *J Pharmacol Exp Ther* 1994; **268**: 868–872.
9. Manning PJ, Rokach J, Malo JL, Ethier D, Cartier A, Girard Y, Charleson S, O'Byrne PM. Urinary leukotriene $E_4$ levels during early and late asthmatic responses. *J Allergy Clin Immunol* 1990; **86**: 211–220.
10. Bernard GR, Korley V, Chee P, Swindell B, Ford-Hutchinson AW, Tagari P. Persistent generation of peptido leukotrienes in patients with the adult respiratory distress syndrome. *Am Rev Respir Dis* 1991; **144**: 263–267.
11. Cook AJ, Yuksel B, Sampson AP, Greenough A, Price JF. Cysteinyl leukotriene involvement in chronic lung disease in premature infants. *Eur Respir J* 1996; **9**: 1907–1912.
12. Lee TH, Crea AEG, Gant V, Spur BW, Marron BE, Nicolaou KC, Reardon E, Brezinski M, Serhan CN. Identification of lipoxin $A_4$ and its relationship to the sulfidopeptide leukotrienes $C_4$, $D_4$ and $E_4$ in the bronchoalveolar lavage fluids obtained from patients with selected pulmonary diseases. *Am Rev Respir Dis* 1990; **141**: 1453–1458.
13. Sampson AP, Green CP, Spencer DA, Piper PJ, Price JF. Leukotrienes in the blood and urine of children with acute asthma. *Ann NY Acad Sci* 1991; **629**: 437–439.
14. Christman BW, McPherson CD, Newman JH, King GA, Bernard GR, Groves BM, Loyd JE. An imbalance between the excretion of thromboxane and prostacyclin metabolites in pulmonary hypertension. *N Engl J Med* 1992; **327**: 70–75.
15. Heavey DJ, Soberman RJ, Lewis RA, Spur B, Austen KF. Critical considerations in the development of an assay for sulfidopeptide leukotrienes in plasma. *Prostaglandins* 1987; **33**: 693–705.
16. Wescott JY, McDonnell TJ, Voekel NF. Alveolar transfer and metabolism of eicosanoids in the rat. *Am Rev Respir Dis* 1989; **139**: 80–87.

ACKNOWLEDGEMENT. The authors wish to thank Dr Serge Demontoux for excellent support during the surgical intervention.

**Received 4 August 1997; accepted in revised form 4 September 1997**
